# Supplementary material for: Migration, hotspots, and dispersal of HIV infection in Rakai, Uganda
Source: Nat Commun. 2020 Feb 20;11:976. doi: 10.1038/s41467-020-14636-y (PMC7033206; doi:10.1038/s41467-020-14636-y)
Supplement: Supplementary file 1 — Supplementary Information [file 41467_2020_14636_MOESM1_ESM.pdf]

Supplementary Information for

Migration, hotspots, and dispersal of HIV infection in Rakai, Uganda

Grabowski, et al.

Supplementary Table 1: RCCS survey participation rates in RCCS R15 and RCCS R16 among long-term residents and in-migrants and out-migrants by age, sex, and place of residence at census.

	RCCS R15 (N=23,415)			RCCS R16 (N=26,084)		
	Long-term residents*	Out-migrants	p-value†	Long-term residents*	In-migrants	p-value†
**Overall**	12596/17830 (71%)	3284/5585 (59%)	<0.001	13186/20586 (64%)	3665/5498 (67%)	<0.001
**Sex**						
Men	5953/9133 (65%)	1422/2637 (54%)	<0.001	6331/10676 (59%)	1450/2445 (59%)	1.0
Women	6643/8697 (76%)	1862/2948 (63%)	<0.001	6855/9910 (69%)	2215/3053 (73%)	<0.001
**Age (years)**						
15-19	1947/3461 (56%)	775/1541 (50%)	<0.001	2313/4566 (51%)	670/1027 (65%)	<0.001
20-24	1927/2785 (69%)	860/1411 (61%)	<0.001	1918/3191 (60%)	1098/1530 (72%)	<0.001
25-29	2442/3340 (73%)	669/1106 (60%)	<0.001	2248/3498 (64%)	799/1233 (65%)	0.52
30-34	2377/3133 (76%)	498/757 (66%)	<0.001	2362/3402 (69%)	530/814 (65%)	0.019
35-39	1937/2525 (77%)	273/428 (64%)	<0.001	2011/2804 (72%)	327/510 (64%)	<0.001
≥40	1966/2586 (76%)	209/342 (61%)	<0.001	2334/3125 (75%)	241/394 (61%)	<0.001
**Place of residence**						
Agrarian	8144/11264 (72%)	1783/2927 (61%)	<0.001	8934/13012 (69%)	1725/2402 (72%)	0.002
Trading	1521/2716 (56%)	562/1211 (46%)	<0.001	1543/3255 (47%)	725/1229 (59%)	<0.001
Fish landing site	2931/3850 (76%)	939/1447 (65%)	<0.001	2709/4319 (63%)	1215/1867 (65%)	0.082

Of the 7535 non-participants in RCCS R15, 7122 (95%) were away for work/school, 167 (2%) refused to participate, and 246 (3%) did not participate for other reasons; Of the 9233 non-participants in RCCS R16, 8910 (97%) were away for work/school, 80 (1%) refused to participate, and 243 (3%) did not participate for other reasons; Long-term residents are considered anyone who was not classified as an out-migrant at RCCS R15 (i.e., moved out of study communities between survey rounds) or an in-migrant at RCCS R16 (i.e. moved into study communities between rounds); † Chi-squared p-value

Supplementary Table 2: Factors associated with successful geocoding* of migrant place of destination/origin

	Out-migrants (N=5,585)		In-migrants (N=5,498)	
	No. successfully geocoded/Total	p-value	No. successfully geocoded /Total	p-value
**Overall**	4122/5585 (74%)		4637/5498 (84%)	
**Sex**				
Men	2196/2948 (74%)		2005/2445 (82%)	
Women	1926/2637 (73%)	0.229	2634/3053 (86%)	<0.001
**Age (years)**				
15-19	1303/1541 (85%)		895/1027 (87%)	
20-24	1047/1411 (74%)		1318/1530 (86%)	
25-29	741/1106 (67%)		1018/1223 (83%)	
30-34	513/757 (68%)		656/814 (81%)	
35-39	291/428 (68%)		419/510 (82%)	
≥40	227/342 (66%)	<0.001	331/394 (84%)	0.001
**Place of residence**				
Agrarian	2375/2927 (81%)		2040/2402 (85%)	
Trading	753/1211 (62%)		1002/1229 (82%)	
Fish landing site	994/1447 (69%)	<0.001	1595/1867 (85%)	0.008
**Reason for Move†**				
Marriage	148/153 (97%)		758/841 (90%)	
Divorced/separated	394/518 (76%)		6/6 (100%)	
Work	1706/1943 (88%)		1909/2209 (86%)	
Education	66/69 (96%)		41/46 (89%)	
Started a new household	535/703 (76%)		480/609 (79%)	
Living with relatives/friends	1167/1435 (81%)		1316/1499 (88%)	
Don't know	103/712 (14%)		2/2 (100%)	
Other	3/3 (100%)	<0.001	125/153 (82%)	<0.001

*Community of destination and community of origin were recorded as free responses at time of census for out-migrants and in-migrants, respectively. These places were geocoded using Google Earth by trained Ugandan investigators. Factors associated with successful geocoding (GPS)/unsuccessful geocoding (NO GPS) are shown in the table. † Information on reason for movement was missing for 49 out-migrants and 133 in-migrants and all of these persons did not have an identifiable place of origin or destination (e.g. GPS data).

Supplementary Table 3a: Female HIV prevalence among in-migrants and out-migrants versus long-term residents at RCCS R15 and R16, respectively.

	Female HIV prevalence among out-migrants and long-term residents at R15					
**Community Type**	**HIV prevalence among long-term residents (HIV positive cases/Total population)**	**HIV prevalence among out-migrants (HIV positive cases/Total population)**	**PRR (95%CI)**	**p-value**	**Age adjusted PRR* (95%CI)**	**p-value**
All Communities	23% (1543/6643)	27% (495/1862)	1.14 (1.03-1.27)	0.009	1.27 (1.14-1.41)	<0.001
Agrarian communities	16% (707/4383)	17% (178/1030)	1.07 (0.90-1.26)	0.41	1.50 (1.26-1.58)	<0.001
Trading Communities	19% (176/896)	21% (70/337)	1.06 (0.80-1.39)	0.69	1.28 (0.95-1.69)	0.095
Fishing Communities	48% (660/1364)	50% (247/495)	1.03 (0.89-1.19)	0.68	1.14 (0.98-1.32)	0.077
	**Female HIV prevalence among in-migrants and long-term residents at R16**					
**Community Type**	**HIV prevalence among long-term residents (HIV positive cases/Total population)**	**HIV prevalence among in-migrants (HIV positive cases/Total population)**	**PRR (95%CI)**	**p-value**	**Age adjusted PRR* (95%CI)**	**p-value**
All Communities	21% (1460/6855)	26% (574/2215)	1.22 (1.10-1.34)	<0.001	1.30 (1.18-1.48)	<0.001
Agrarian communities	15% (712/4711)	19% (212/1115)	1.26 (1.08-1.46)	0.003	1.64 (1.39-1.92)	<0.001
Trading Communities	19% (172/909)	18% (83/472)	0.93 (0.71-1.20)	0.58	1.25 (0.95-1.65)	0.11
Fishing Communities	47% (576/1235)	44% (279/628)	0.95 (0.82-1.10)	0.51	1.08 (0.93-1.26)	0.29

PRR=Prevalence Risk ratio; 95% CI= 95% confidence interval; RCCS=Rakai Community Cohort Study; adjPRR=adjusted PRR; *Overall analysis for all communities adjusted for age and community-type.

Supplementary Table 3b: Male HIV prevalence among in-migrants and out migrants versus long-term residents at RCCS R15 and R16, respectively.

	Male HIV prevalence among out-migrants and long-term residents at R15					
**Community Type**	**HIV prevalence among long-term residents (HIV positive cases/Total population)**	**HIV prevalence among out-migrants (HIV positive cases/Total population)**	**PRR (95%CI)**	**p-value**	**Age adjusted PRR* (95%CI)**	**p-value**
All Communities	18% (1050/5953)	16% (223/1422)	0.89 (0.77-1.03)	0.11	0.96 (0.82-1.11)	0.56
Agrarian communities	11% (414/3761)	10% (72/753)	0.87 (0.67-1.11)	0.27	1.17 (0.89-1.49)	0.23
Trading Communities	12% (74/625)	10% (22/225)	0.83 (0.50-1.31)	0.43	1.04 (0.63-1.66)	0.86
Fishing Communities	36% (562/1567)	29% (129/444)	0.81 (0.67-0.98)	0.031	0.86 (0.71-1.04)	0.14
	**Male HIV prevalence among in-migrants and long-term residents at R16**					
**Community Type**	**HIV prevalence among long-term residents (HIV positive cases/Total population)**	**HIV prevalence among in-migrants (HIV positive cases/Total population)**	**PRR (95%CI)**	**p-value**	**Age adjusted PRR* (95%CI)**	**p-value**
All Communities	16% (1014/6331)	16% (230/1450)	0.99 (0.86-1.14)	0.90	0.90 (0.78-1.04)	0.16
Agrarian communities	10% (432/4223)	10% (59/610)	0.95 (0.73-1.23)	0.69	1.06 (0.80-1.39)	0.69
Trading Communities	10% (65/634)	9% (23/253)	0.89 (0.54-1.40)	0.62	1.10 (0.66-1.77)	0.70
Fishing Communities	35% (517/1474)	25% (148/587)	0.72 (0.59-0.86)	<0.001	0.81 (0.67-0.98)	0.029

PRR=Prevalence Risk ratio; 95% CI= 95% confidence interval; RCCS=Rakai Community Cohort Study; adjPRR=adjusted PRR;
*Overall analysis for all communities adjusted for age and community-type.

Supplementary Table 4: Prevalence risk ratios comparing HIV prevalence among in-migrants at R16 vs. HIV prevalence among out-migrants at R15 stratified by community type and sex.

HIV prevalence among female in-migrants at R16 vs. HIV prevalence among female out-migrants at R15

Community Type	PRR (95%CI)	p-value	Age adjusted PRR* (95%CI)	p-value
All Communities	0.97 (0.86-1.10)	0.68	0.95 (0.84-1.07)	0.42
Agrarian communities	1.10 (0.90-1.34)	0.35	1.03 (0.84-1.26)	0.78
Trading Communities	0.85 (0.61-1.17)	0.31	0.89 (0.65-1.23)	0.50
Fishing Communities	0.89 (0.75-1.06)	0.18	0.89 (0.75-1.06)	0.18

HIV prevalence among male in-migrants at R16 vs. HIV prevalence among male out-migrants at R15

Community Type	PRR (95%CI)	p-value	Age adjusted PRR* (95%CI)	p-value
All Communities	1.01 (0.84-1.22)	0.90	0.94 (0.78-1.13)	0.51
Agrarian communities	1.01 (0.71-1.43)	0.95	0.85 (0.60-1.20)	0.35
Trading Communities	0.93 (0.52-1.68)	0.81	1.07 (0.59-1.94)	0.83
Fishing Communities	0.87 (0.69-1.10)	0.24	0.89 (0.70-1.12)	0.31

PRR=Prevalence Risk ratio; 95% CI= 95% confidence interval; RCCS=Rakai Community Cohort Study; adjPRR=adjusted PRR; *Overall analysis for all communities adjusted for age and community-type.

Supplementary Table 5: Inverse probability weighted relative prevalence of self-reported ART use among in-migrants and out-migrants vs. long-term residents by age and sex.

	**HIV-positive women (N=2038), RCCS R15**				**HIV-positive women (N=2034), RCCS R16**			
Age (years)	Out-migrant vs. Long-term resident, PRR_w (95%CI)	p-value	Out-migrant vs. Long-term resident, adjPRR_w (95%CI)	p-value	In-migrant vs. Long-term resident, PRR_w (95%CI)	p-value	In-migrant vs. Long-term resident, adjPRR_w (95%CI)	p-value
15-24	0.86 (0.47-1.56)	0.382	0.77 (0.43-1.38)	0.382	0.55 (0.40-0.75)	<0.001	0.57 (0.42-0.78)	<0.001
25-35	0.84 (0.63-1.12)	0.248	0.84 (0.63-1.11)	0.221	0.68 (0.58-0.81)	<0.001	0.68 (0.57-0.80)	<0.001
35-49	0.73 (0.55-0.98)	0.038	0.79 (0.59-1.11)	0.103	0.71 (0.59-0.86)	<0.001	0.73 (0.60-0.88)	<0.001
All	0.65 (0.53-0.79)	<0.001	0.83 (0.69-1.01)	0.063	0.59 (0.52-0.66)	<0.001	0.67 (0.59-0.76)	<0.001
	**HIV-positive men (N=1273), RCCS R15**				**HIV-positive men (N=1244), RCCS R16**			
Age (years)	Out-migrant vs. Long-term resident, PRR_w (95%CI)	p-value	Out-migrant vs. Long-term resident, adjPRR_w (95%CI)	p-value	In-migrant vs. Long-term resident, PRR_w (95%CI)	p-value	In-migrant vs. Long-term resident, adjPRR_w (95%CI)	p-value
15-24	0.37 (0.05-2.79)	0.362	0.41 (0.06-3.00)	0.383	0.86 (0.24-3.11)	0.813	0.83 (0.25-2.72)	0.761
25-35	0.61 (0.33-1.12)	0.110	0.58 (0.32-1.08)	0.088	0.77 (0.55-1.09)	0.139	0.80 (0.57-1.12)	0.196
35-49	0.63 (0.38-1.05)	0.074	0.63 (0.40-1.07)	0.090	0.68 (0.50-0.92)	0.012	0.69 (0.51-0.93)	0.017
All	0.53 (0.35-0.78)	0.001	0.59 (0.40-0.87)	0.007	0.63 (0.50-0.79)	<0.001	0.75 (0.59-0.94)	0.014

PRR=Prevalence Risk ratio; PRR_w=Prevalence Risk ratio; 95% CI= 95% confidence interval; RCCS=Rakai Community Cohort Study; adjPRR=adjusted PRR; *Age-stratified analyses adjusted for community type. Overall analysis for all communities adjusted for age and community-type.

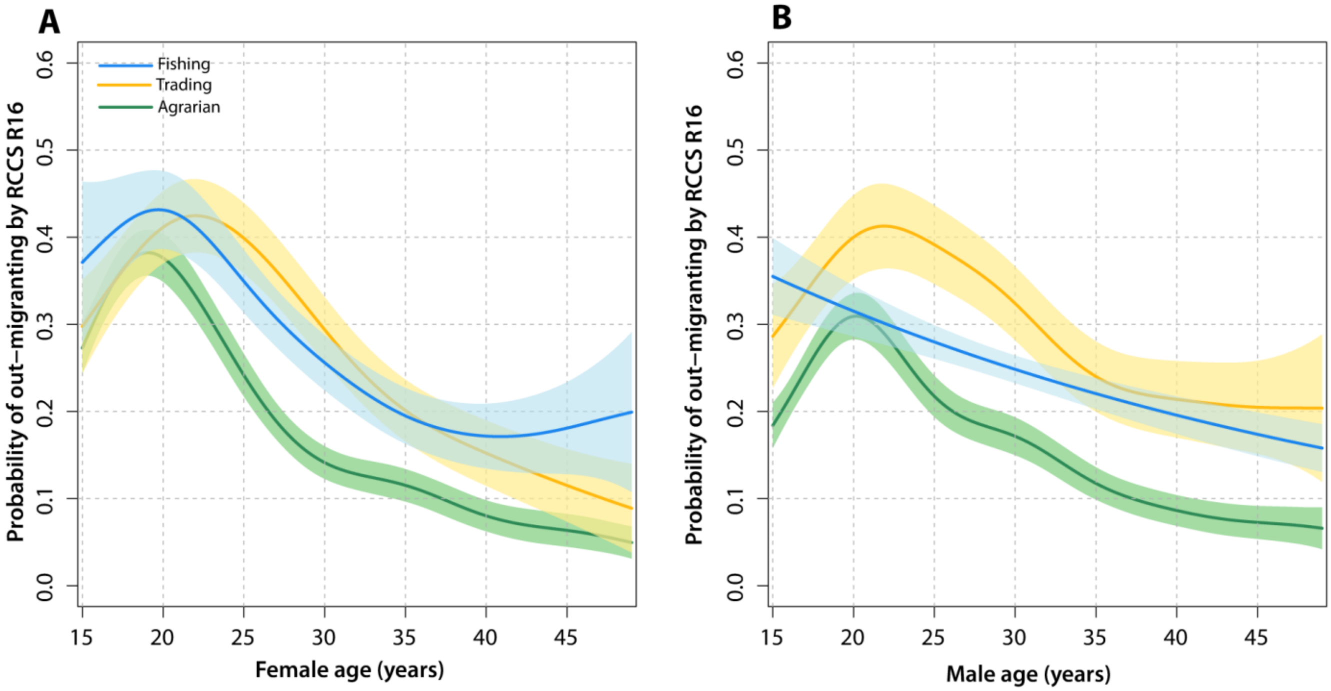

Supplementary Figure 1. Proportion of women (A) and men (B) at R15 being classified as an out-migrant by R16 by age and community-type with 95% confidence intervals shown in shaded areas.

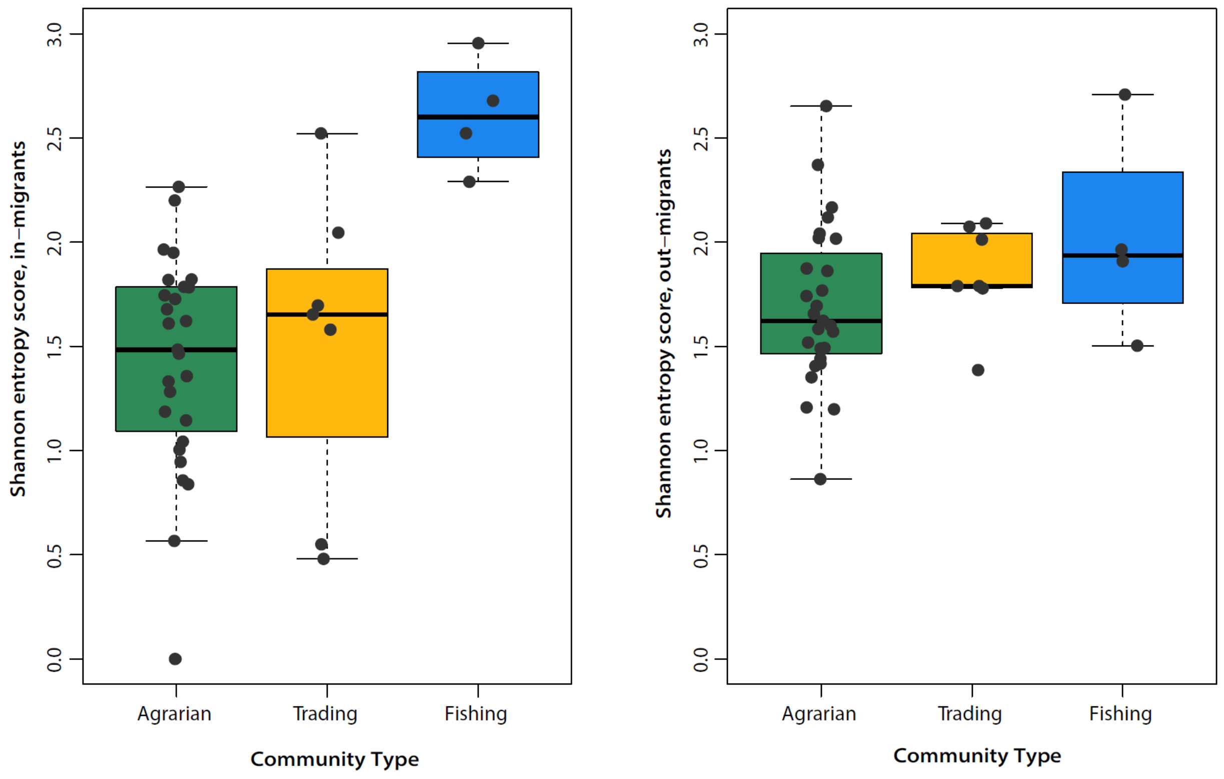

Supplementary Figure 2: Boxplot of community-level Shannon entropy scores of the geographic diversity of source/destination locations measured at the district level (i.e., administrative unit 1) among in-migrants and out-migrants in RCCS agrarian, trading, and fishing communities. The bottom and top of the boxes represent the 25th and 75th percentiles of the data (i.e., interquartile range), respectively. The median (50th percentile) is indicated in the middle of the box with a solid black line. Individual data points, representing each community's Shannon entropy score, are shown as dark gray circles. Box limits (whiskers) are plotted at 1.5 times the interquartile range.

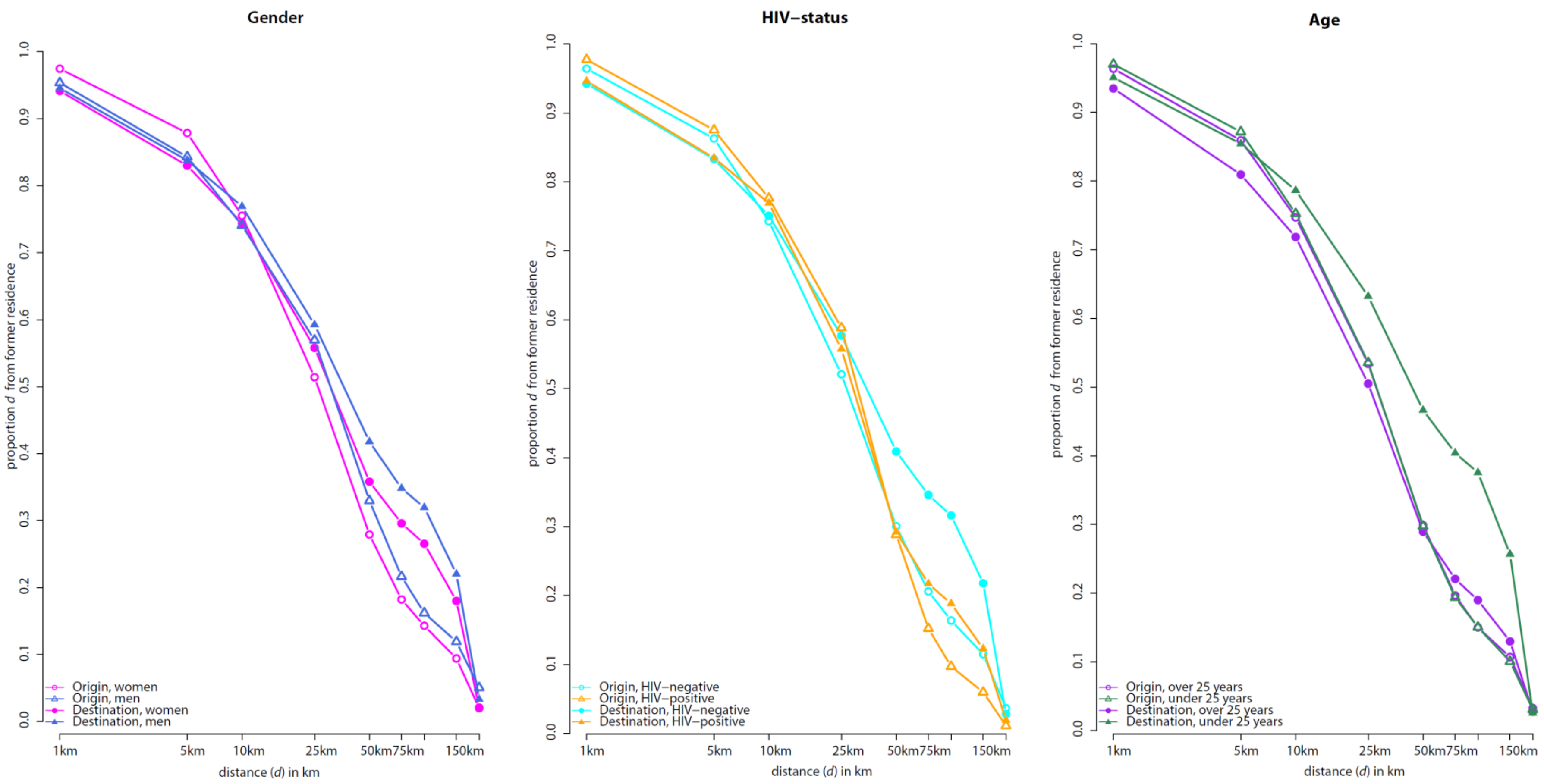

Supplemental Figure 3. Inverse cumulative distance kernels for place of origin and destination for in-migrants and out migrants, respectively. Figure shows proportion who migrated at (or further) particular distances from home. Distances (*d*) are from the source/destination location of each participant to their current/former household in the RCCS in kilometers. Analyses are stratified by gender, HIV-status and age.
