# Supplementary material for: Migration, hotspots, and dispersal of HIV infection in Rakai, Uganda
Source: Nat Commun. 2020 Feb 20;11:976. doi: 10.1038/s41467-020-14636-y (PMC7033206; doi:10.1038/s41467-020-14636-y)
Supplement: Supplementary file 3 — Reporting Summary [file 41467_2020_14636_MOESM3_ESM.pdf]

natureresearch

Corresponding author(s): Mary K. Grabowski

Last updated by author(s): Jan 6, 2020

# Reporting Summary

Nature Research wishes to improve the reproducibility of the work that we publish. This form provides structure for consistency and transparency in reporting. For further information on Nature Research policies, see Authors & Referees and the Editorial Policy Checklist .

## Statistics

For all statistical analyses, confirm that the following items are present in the figure legend, table legend, main text, or Methods section.

| n/a | Confirmed | |
|---|---|---|
| ☐ | ☒ | The exact sample size ($n$) for each experimental group/condition, given as a discrete number and unit of measurement |
| ☐ | ☒ | A statement on whether measurements were taken from distinct samples or whether the same sample was measured repeatedly |
| ☐ | ☒ | The statistical test(s) used AND whether they are one- or two-sided<br>*Only common tests should be described solely by name; describe more complex techniques in the Methods section.* |
| ☐ | ☒ | A description of all covariates tested |
| ☐ | ☒ | A description of any assumptions or corrections, such as tests of normality and adjustment for multiple comparisons |
| ☐ | ☒ | A full description of the statistical parameters including central tendency (e.g. means) or other basic estimates (e.g. regression coefficient) AND variation (e.g. standard deviation) or associated estimates of uncertainty (e.g. confidence intervals) |
| ☒ | ☐ | For null hypothesis testing, the test statistic (e.g. $F$, $t$, $r$) with confidence intervals, effect sizes, degrees of freedom and $P$ value noted<br>*Give P values as exact values whenever suitable.* |
| ☒ | ☐ | For Bayesian analysis, information on the choice of priors and Markov chain Monte Carlo settings |
| ☒ | ☐ | For hierarchical and complex designs, identification of the appropriate level for tests and full reporting of outcomes |
| ☐ | ☒ | Estimates of effect sizes (e.g. Cohen's $d$, Pearson's $r$), indicating how they were calculated |

*Our web collection on statistics for biologists contains articles on many of the points above.*

## Software and code

Policy information about availability of computer code

| | |
|---|---|
| Data collection | All code and data is available on github as indicated in the manuscript. |
| Data analysis | R version 3.6.1 was used to conduct all data analyses. Code to reproduce this manuscript's figures is available through Github (https://github.com/HopkinsIDD) and published on Zenodo (DOI: 10.5281/zenodo.3533472). |

For manuscripts utilizing custom algorithms or software that are central to the research but not yet described in published literature, software must be made available to editors/reviewers. We strongly encourage code deposition in a community repository (e.g. GitHub). See the Nature Research guidelines for submitting code & software for further information.

## Data

Policy information about availability of data

All manuscripts must include a data availability statement. This statement should provide the following information, where applicable:

- Accession codes, unique identifiers, or web links for publicly available datasets
- A list of figures that have associated raw data
- A description of any restrictions on data availability

The data used to conduct these analyses include personally identifying geographic and health information protected by privacy law and the Institutional Review Boards (IRB) that approved this study. Individual-level data is available upon request to the Rakai Health Sciences Program (www.rhsp.org) and following IRB approval of investigators and analysis protocols. Partial de-identified datasets including basic aggregate-level epidemiological data on key variables (sex, age group, gender, migration status, HIV status, self-reported ART use, and community type) and matrices used to construct network figures are available are available through Github (https://github.com/HopkinsIDD)) and published on Zenodo (DOI: 10.5281/zenodo.3533472).

# Field-specific reporting

Please select the one below that is the best fit for your research. If you are not sure, read the appropriate sections before making your selection.

☐ Life sciences ☒ Behavioural & social sciences ☐ Ecological, evolutionary & environmental sciences

For a reference copy of the document with all sections, see nature.com/documents/nr-reporting-summary-flat.pdf

# Behavioural & social sciences study design

All studies must disclose on these points even when the disclosure is negative.

| | |
|---|---|
| Study description | Observational population-based cohort study of HIV infection |
| Research sample | We used data from the Rakai Community Cohort Study (RCCS), an open population-based census and cohort. Individuals, including migrants, can enter or exit the study population between surveys. The RCCS surveys individuals aged 15-49 in 40 communities in and near the predominately rural Rakai District of south central Uganda. We used data collected from two RCCS surveys designated as R15 (August 8, 2011-May 30, 2013) and R16 (August 21, 2014-January 30, 2015) from 38 continuously surveyed communities. |
| Sampling strategy | Population-based sample of all residents living in 38 communities between the ages of 15-49 with capacity for consent. |
| Data collection | The RCCS conducts a household census with no age truncation prior to the cohort survey. Census data include household GPS location and information on each household member including name, age, gender, marital status, and familial and marital relationships. Individuals who have migrated into or out of a household since the prior census are identified. The RCCS survey, conducted after the census, includes all consenting residents aged 15-49. Interviewers by same sex interviewers use structured questionnaires in the local language (Luganda) to collect sociodemographic, behavioral, and health information. Data are directly entered into mobile PCs and edited in the field. HIV testing is performed using a validated three rapid test algorithm. Pre- and post-test counseling and HIV test results are offered by on-site counselors at time of survey. |
| Timing | August 2011-January 2015 |
| Data exclusions | No data was excluded |
| Non-participation | Data on non-participation and missing data is summarized in the results and Supplemental Tables 1 and 2. |
| Randomization | N/A |

# Reporting for specific materials, systems and methods

We require information from authors about some types of materials, experimental systems and methods used in many studies. Here, indicate whether each material, system or method listed is relevant to your study. If you are not sure if a list item applies to your research, read the appropriate section before selecting a response.

## Materials & experimental systems

| n/a | Involved in the study | |
|---|---|---|
| ☒ | ☐ | Antibodies |
| ☒ | ☐ | Eukaryotic cell lines |
| ☒ | ☐ | Palaeontology |
| ☒ | ☐ | Animals and other organisms |
| ☐ | ☒ | Human research participants |
| ☒ | ☐ | Clinical data |

## Methods

| n/a | Involved in the study | |
|---|---|---|
| ☒ | ☐ | ChIP-seq |
| ☒ | ☐ | Flow cytometry |
| ☒ | ☐ | MRI-based neuroimaging |

## Human research participants

Policy information about studies involving human research participants

| | |
|---|---|
| Population characteristics | This information is included in Table 1 and Results section |
| Recruitment | Eligible individuals 15-49 are recruited through a household census as described in the data collection section above. |
| Ethics oversight | Uganda Virus Research Institute, Uganda; Western IRB |

Note that full information on the approval of the study protocol must also be provided in the manuscript.
